# Immunogenicity of autoantigens

**DOI:** 10.1186/1471-2164-12-340

**Published:** 2011-07-04

**Authors:** Christina Backes, Nicole Ludwig, Petra Leidinger, Christian Harz, Jana Hoffmann, Andreas Keller, Eckart Meese, Hans-Peter Lenhof

**Affiliations:** 1 Center for Bioinformatics, Saarland University, 66041 Saarbrücken, Germany; 2 Department of Human Genetics, Saarland University, 66421 Homburg/Saar, Germany

## Abstract

**Background:**

Autoantibodies against self-antigens have been associated not only with autoimmune diseases, but also with cancer and are even found in healthy individuals. The mechanism causing the autoantibody response remains elusive for the majority of the immunogenic antigens. To deepen the understanding of autoantibody responses, we ask whether natural-occurring, autoimmunity-associated and tumor-associated antigens have structural or biological features related to the immune response. To this end, we have carried out the most comprehensive in-silicio study of different groups of autoantigens including large antigen sets identified by our groups combined with publicly available antigen sets.

**Results:**

We found evidence for an enrichment of genes with a larger exon length increasing the probability of the occurrence of potential immunogenic features such as mutations, SNPs, immunogenic sequence patterns and structural epitopes, or alternative splicing events. While SNPs seem to play a more central role in autoimmunity, somatic mutations seem to be stronger enriched in tumor-associated antigens. In addition, antigens of autoimmune diseases are different from other antigen sets in that they appear preferentially secreted, have frequently an extracellular location, and they are enriched in pathways associated with the immune system. Furthermore, for autoantibodies in general, we found enrichment of sequence-based properties including coiled-coils motifs, ELR motifs, and Zinc finger DNA-binding motifs. Moreover, we found enrichment of proteins binding to proteins or nucleic acids including RNA and enrichment of proteins that are part of ribosome or spliceosome. Both, homologies to proteins of other species and an enrichment of ancient protein domains indicate that immunogenic proteins are evolutionary conserved and that mimicry might play a central role.

**Conclusions:**

Our results provide evidence that proteins which i) are evolutionary conserved, ii) show specific sequence motifs, and iii) are part of cellular structures show an increased likelihood to become autoimmunogenic.

## Background

The generation of autoantibodies against self-antigens is a common phenomenon in humans. Autoantibodies have been directly associated with the pathophysiology of some diseases most prominently with autoimmune diseases. They also appear to occur in the context of many other diseases as cancer or have even been reported in apparently healthy individuals. The meaning of these autoantibodies is not understood and especially the underlying mechanism eliciting an autoantibody response remains elusive for the majority of the immunogenic antigens. For the purpose of systematization, we differentiate between natural-occurring, autoimmunity-associated and tumor-associated antigens (HAGs, AAGs and TAGs, respectively). One has to be aware that this grouping may be somewhat arbitrary since many antigens seem to appear in more than one of the proposed groups. Instead of allocating single antigens to a specific group of diseases and even to a specific disease, it appears more appropriate to allocate seroreactivity patterns, e.g. the reactivity of multiple autoantibodies to a disease or group of diseases. This idea is strongly supported by identification of autoantibody reactivity patterns, also addressed as autoantibody signatures that are highly specific for various cancers and some non-cancer diseases as shown by us and others [1-10]. Provided that the identity of tumor associated antigens mirrors deregulated pathways of a specific cancer as recently suggested [11], autoantibody signatures against tumor antigens might provide insight into many of the deregulated pathways in cancer.

There are multiple reasons proposed why self-proteins become immunogenic including mutations, alternative splicing, post-translational modifications, deregulated apoptotic or necrotic processes, expression of fetal proteins in adult tissue, single nucleotide polymorphisms (SNPs), differential cellular localization and overexpression [12-14]. For almost all antigens, the underlying reason for their immunogenicity has not yet been elucidated. The cause of immunogenicity may be rather complex as shown for the tumor suppressor protein p53. Mutations in p53 have been identified for several cancers and other diseases. In patients with rheumatoid arthritis, somatic mutations of p53 are commonly found, whereas p53 antibodies were only rarely detected in sera or synovial fluids [15]. In contrast, autoantibodies against p53 are detected in 4 - 30% of sera of patients with various types of cancers [16], but only 20-40% of the patients with p53 mutations have autoantibodies against p53. These autoantibodies recognize both mutated and wild-type p53 [17]. While the mutations are located in the central protein, the epitopes of the anti-p53 antibodies mostly map in the highly glycosylated amino- and carboxy-terminal ends of the protein [18]. The mutation causes a structural change in the p53 protein that results in an increased half-life of the protein. The prolonged half-life of the mutated protein and the resulting accumulation of p53 in the cell is likely a prerequisite for its immunogenicity rather than the structural changes caused by the mutation itself.

Besides mutations, the overexpression of fetal proteins in cancer might also elicit an immune response. This is due to the absence of fetal proteins during the time, when the immune system is developing tolerance against self-antigens. This seems to hold true for the cancer-testis antigen NY-ESO-1, that was originally identified as TAG in esophageal squamous cell carcinoma. It is also expressed in several other cancers, e.g. breast cancer, melanoma or prostate cancer [19].

Another interesting hypothesis is that alterated alternative splicing might play a central role in autoimmunity. Ng et al. analyzed the extent of alternative splicing in known self-proteins with an association to autoimmune diseases and compared them to randomly selected human proteins [14]. They found an increased amount of alternative splicing in the autoantigen transcripts and in addition an increased noncanonical alternative splicing, leading to the hypothesis that the generation of untolerized epitopes may be responsible for the autoimmune response. The proposed "stimulation-responsive splicing" model [20] refined this hypothesis by illustrating how alternative splicing of autoantigen and self-tumor antigen mRNAs in response to stimuli may lead to aberrant expression of antigen isoforms that present novel untolerized epitopes generated by inclusion or depletion of exons.

Alteration in the processes of apoptosis and necrosis can also be associated with the generation of autoantibodies. During apoptosis, antigen clusters are found in apoptotic blebs, which are believed to be a major source of intracellular autoantigens in autoimmune diseases [21]. Some autoimmune diseases, e.g. systemic lupus erythematosus (SLE), show an impaired uptake of apoptotic cells into macrophages resulting in an accumulation of dying cells [22]. These cells release so-called danger signals that can trigger an increased immune response [23]. Due to their prolonged presence in the system, these cells might undergo secondary processes including secondary necrosis as well as massive cell-death related modifications. Antigens may be affected by oxidation, hyperphosphorylation or aberrant cleavage, e.g. through caspases and granzyme B [24-27].

Two processes have to be taken into account when elucidating common features of autoantigens, epitope spreading and molecular mimicry. Epitope spreading refers to a model where an immune response is initiated through an immunodominant epitope, while later on the response expands to other neighboring epitopes in the same protein [28,29]. This model has been confirmed for autoimmune diseases as well as for animal models of immunization [30]. Molecular mimicry describes a mechanism in which structurally-related epitopes in different molecules induce the generation of cross-reactive antibodies as described for autoimmune diseases that were triggered by pathogen infection [31]. Molecular mimicry can be perceived as a form of intermolecular epitope spreading [32].

The present data strongly indicate that autoantigens become immunogenic for various reasons in different diseases. The generation of autoantibody repertoires is a multifactor process involving aberrant expression of proteins or protein structures combined with aberrant cell death procedures and clearance of these cells by the immune system in a danger signal enriched microenvironment. As further step towards a better understanding, we ask whether natural-occurring, autoimmunity-associated and tumor-associated antigens have common structural or biological features and which features differ between these groups. To this end, we performed a comprehensive analysis by compiling data on autoantigens from our own experimental works and from the literature. We analyzed common and differential features using our recently developed GeneTrail [33] analysis tool.

## Results

### Autoantigens possess in general a longer exon length

**Figure 1:**
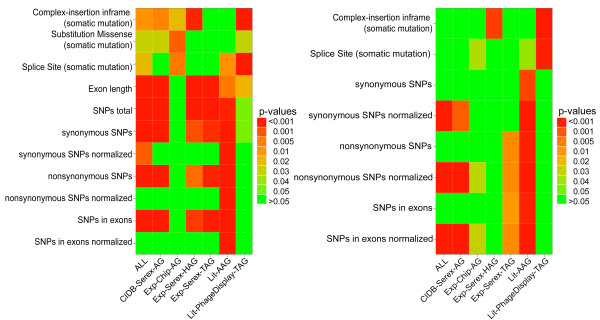
**Heat maps showing the most frequently enriched somatic mutation and SNP categories and the general exon length in the considered data sets**. Heat maps showing the most frequently enriched somatic mutation and SNP categories and the general exon length in the considered data sets compared to the ProteinCodingGenes reference set (left-hand side) or compared to the ProteinCodingGenesLongerExons reference set (right-hand side). ALL: union of all antigen sets; CIDB-Serex-AG: retrieved from the Cancer Immunome Database (SEREX method); Exp-Chip-AG: mixed antigens (tumor/non-tumor diseases) identified by protein macroarray; Exp-Serex-HAG: natural occurring autoantigens (SEREX method); Exp-Serex-TAG: tumor antigens (SEREX method); Lit-AAG: autoimmune antigens collected by literature search; Lit-PhageDisplay-TAG: tumor antigens identified by Phage Display experiments collected by literature search

We compared the mean exon length of the genes in our seven test sets with the reference set termed ProteinCodingGenes (PCG) that incorporates all human protein coding genes excluding the ones contained in the seven test sets. All seven test sets had a larger mean exon length (see Table 1), six had also a significantly longer mean exon length (see Figure 1, left-hand side, row 4 of the matrix). This astonishing observation seems to be plausible at the second sight, because genes with longer exons are transcribed into larger proteins increasing the probability of the occurrence of potential immunogenic features such as mutations, SNPs, immunogenic sequence patterns and structural epitopes. In addition, a larger number of exons provides the possibilty of expressing different splicing products increasing the probability of confronting the immune system with untolerized epitopes. However, the differences in exon length complicate the analysis of these immunogenic features. To solve this problem, we have defined a second reference set called ProteinCodingGenesLongerExons (PCGLE) that contains all genes in PCG with mean exon length greater than 3100 nucleotides. The PCGLE reference set has a mean exon length of 5842 nucleotides that is significantly larger than the mean length of five test sets (ALL: 4906, CIDB-Serex-AG: 5179, Ex-Chip-AG: 4355, Lit-AGG: 4710, Lit-PhageDisplay-TAG: 4782) and that does not differ significantly from the mean of the remaining two small test sets (Exp-Serex-TAG: 6266, Exp-Serex-HAG: 5986) (see Table 1). When considering length dependent parameters, we wanted to exclude an exon length bias and we focussed the discussion here on the results for the five test sets with shorter mean exon length that involve the larger and probably more informative test sets. Please note that significant results for one of these five test sets and length dependent features in comparison with the second reference set reveal a 'significantly' stronger density of the considered features in the test sets. We have chosen this conservative approach that may lead to the loss of some signals, because we did not want to carry out the analyses with different 'random' reference sets for the different test sets. We carried out all analyses with both reference sets PCG and PCGLE and compared the two results whenever we discuss features that may depend on the mean exon length.

**Table 1:** Data sets for our analyses.

Data set	# known genes	mean exon length (nt)	References/Description
CIDB-Serex-AG	1471	5179	http://ludwig-sun5.unil.ch/CancerImmunomeDB/
Exp-Serex-HAG	85	6266	collected by Prof. Meese's group
Exp-Serex-TAG	74	5986	collected by Prof. Meese's group
Exp-Chip-AG	298	4355	collected by Prof. Meese's group
Lit-PhageDisplay-TAG	84	4782	collected by Prof. Meese's group
Lit-AAG	348	4710	http://www.wiley-vch.de/contents/jc_2040/2005/25481_s.pdf
ALL	2079	4906	union of the above antigen sets
ProteinCodingGenes	23583	3812	all protein coding genes retrieved from NCB1 excluding the above antigens
ProteinCodingGenesLongerExons	8816	5842	corresponds to ProteinCodingGenes with genes having exon lengths > 3100 nucleotides

These data sets are also available online: http://genetrail.bioinf.uni-sb.de/paper/ags/

### Somatic mutations seem to be more prevalent in TAGs, SNPs are primarily enriched in AAGs

To test whether our antigen sets have a higher number of somatic mutations or SNPs compared to the two reference sets we extracted mutation and SNP data from current databases and performed Wilcoxon-Mann-Whitney tests (WMW). As source of mutation data we used the 'Roche Cancer Genome Database' (RCGDB) http://rcgdb.bioinf.uni-sb.de/MutomeWeb/ [34] that combines different sources of human mutation databases including the Catalogue of Somatic Mutations in Cancer (COSMIC), the Cancer Genome Atlas, and Online Mendelian Inheritance in Man (OMIM). We extracted for each gene in this database the different types of somatic mutations and the number of their occurrences in cancer. For the SNP data we used the dbSNP database from NCBI [35] as source and extracted the different SNPs for every gene as deposited. The results of the analyses are summarized in Figure 1. The heat map on the left-hand side shows the results for the first reference set PCG and the one on the right-hand side the results for the second reference set PCGLE.

When considering SNPs the most obvious enrichment was found in the set of autoimmune-associated antigens (Lit-AAG). This enrichment was detected for all SNP types, including (non)-synonymous SNPs and (non)-synonymous SNPs normalized. This observation is in line with published data also reporting an enrichment of SNPs in autoantigens associated with autoimmune diseases [36]. For tumor antigens, we obtained ambivalent results. While the test sets CIDB-Serex-AG retrieved form the CIDB database (Cancer Immunome Database) and Exp-Serex-TAG identified by SEREX (Serological Analysis of Recombinant cDNA Expression Libraries) show an enrichment of SNPs, the set Lit-PhageDisplay-TAG was not enriched with any type of SNP. For the Exp-Chip-AG, we did not find an enrichment compared with the first reference set PCG, however, compared with the second reference set PCGLE, we detected an week enrichment of non-synonymous SNPs (normalized), which indicates that this set of tumor antigens has a higher density of non-synonymous SNPs than the reference set.

For somatic mutations, the results of the different test sets are also ambivalent. The comparison with the first reference set PCG indicates that somatic mutations may play a central role for tumor antigens. However, this analysis may only reveal the trivial fact that genes with longer exons may have more (somatic) mutations and hence does not provide strong evidence that somatic mutations may induce the humoral immune reactions. The comparison with the second reference set provides one remarkable result for the two test sets that have been collected by literature search. The comparison of the two sets Lit-AAG and Lit-PhageDisplay indicates that somatic mutations may play a more crucial role for tumor antigens whereas SNPs may play a more central role for autoantigens associated with autoimmune diseases. However, this may be due to the phage display technique that has been used to identify the tumor antigens.

### Autoantigens show in general an enrichment for Granzyme B cleavage sites, coiled-coil motifs, and ELR motifs

**Figure 2:**
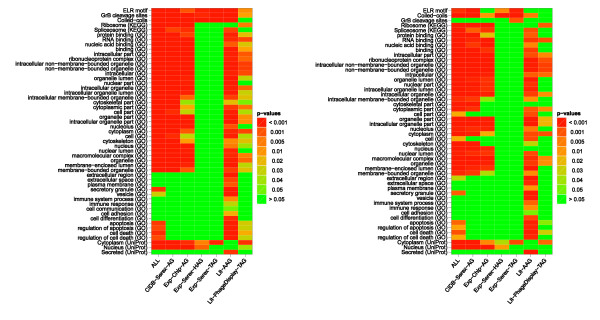
**Heat maps showing the significantly enriched motifs, pathways, GO terms and subcellular locations in the considered data sets**. Heat maps showing the significantly enriched motifs, pathways, GO terms and subcellular locations in the considered data sets compared to the ProteinCodingGenes reference set (left-hand side) or compared to the ProteinCodingGenesLongerExons reference set (right-hand side). ALL: union of all antigen sets; CIDB-Serex-AG: retrieved from the Cancer Immunome Database (SEREX method); Exp-Chip-AG: mixed antigens (tumor/non-tumor diseases) identified by protein macroarray; Exp-Serex-HAG: natural occurring autoantigens (SEREX method); Exp-Serex-TAG: tumor antigens (SEREX method); Lit-AAG: autoimmune antigens collected by literature search; Lit-PhageDisplay-TAG: tumor antigens identified by Phage Display experiments collected by literature search

To analyze sequence-based properties of antigens, we focused on Granzyme B (GrB) cleavage sites [37], coiled-coils [38], and ELR motifs (Glu-Leu-Arg) [39], all of which were previously associated with immunogenic antigens. The analysis with reference PCG shows that Granzyme B cleavage sites and ELR motifs were enriched in all data sets. Coiled-coils were also enriched in all data sets except for tumor antigens identified by phage display (Lit-PhageDisplay-TAG). These data are largely in agreement with the idea that these sequence motifs play a role in the immunogenicity of autoantigens. The comparison with the second reference set PCGLE confirms these results basically for ELR motifs and coiled-coils, but not for Granzyme B cleavage sites. However, it is important to bear in mind that Granzyme B cleavage sites and coiled-coils are predicted and not necessarily experimentally proven features. The results are summarized in the heat maps of Figure 2.

### Autoantigens are associated with binding functions

In addition to sequence-based properties, we analyzed whether certain functional groups, processes or subcellular locations play a role in the immunogenicity of antigens. To his end, we utilized Gene Ontology (GO) [40]. The GO hierarchy consists of three main categories: molecular function, cellular component, and biological process. For the ORA of GO terms, we used only manually curated GO annotations and not the computationally assigned annotations (with 'IEA' (Inferred from Electronic Annotation) evidence code). Since this analysis yielded more than 200 subcategories that were significant in at least one antigen set, in the following we focus only on the most interesting results for the GO hierarchies molecular function and cellular component. Most notably, we found in most antigen sets an enrichment for the category binding and related categories including protein binding, nucleic acid binding, and RNA binding. These results provide first evidence that proteins that are part of cellular structures may show an increased likelihood to become immunogenic. Lack of enrichment for most of these categories was observed for the two data sets Exp-Serex-HAG and Exp-Serex-TAG derived with the SEREX method (Figure 2).

### AAGs are often extracellularly localized or get secreted

Another interesting result of the GO analysis showed that antigens identified in autoimmune diseases (Lit-AAG) are enriched for GO terms that are not enriched in other data sets. These GO terms belong to 'extracellular region/space', 'plasma membrane', 'vesicle', and 'secretory granule'. These findings indicate that antigens of autoimmune diseases have the tendency to get secreted and/or have an extracellular location where they might more readily stimulate an immune reaction. These results are also summarized in Figure 2. Furthermore, antigens identified in autoimmune diseases (Lit-AAG) are enriched for manycategories related to the immune system and apoptosis. The complete list of all 222 significantly enriched subcategories is supplied in Additional file 1 Figure S1. In addition to the GO annotations derived from the 'cellular component' hierarchy, we used the subcellular locations annotated from UniProt as a second data source to test whether the antigens are enriched in a specific subcellular location [41]. We extracted the corresponding information from the UniProtKB/Swiss-Prot flatfile ftp://ftp.uniprot.org/pub/databases/uniprot/current_release/knowledgebase/complete/uniprot_sprot.dat.gz and performed an ORA of all antigen data sets using the ProteinCodingGenes as reference. The Lit-AAG set was enriched for the category 'Secreted' only. With exception of 'Exp-Serex-TAG' all other antigen sets were enriched for the categories 'Nucleus' and 'Cytoplasm'.

### AAGs are associated with immune system pathways

To explore if our antigen sets have certain pathways in common and if these pathways are involved in immunogenic processes, we used the KEGG (Kyoto Encyclopedia of Genes and Genomes) database, a comprehensive repository containing regulatory as well as metabolic pathways [42]. We found enrichment for the subcategories 'Ribosome' and 'Spliceosome'. These results provide further evidence that proteins, being part of cellular structures have a propensity to become immunogenic. As above, the enrichment of the subcategories Ribosome and Spliceosome was not found for tumor antigens and antigens found in healthy persons, both of which were identified by a SEREX screening. The latter antigen group also did not show enrichment for any of the pathway categories. The other antigen groups showed sporadic enrichment for a few metabolic pathways and many regulatory, signal-transduction and cancer pathways (see Additional file 2 Figure S2). As previously observed, antigens of autoimmune diseases are different from other antigen sets in that they show enriched pathways that are not detected for any other antigen set. These are pathways of the immune system including 'Complement and coagulation cascades', 'Antigen processing and presentation', 'Hematopoietic cell lineage', 'ECM-receptor interaction', the 'Jak-STAT signaling pathway', and the autoimmune disease 'Systemic lupus erythematosus'. This observation suggests that the occurrence of self-antigens in autoimmune diseases results from mechanisms that are different from the mechanisms occurring in cancer and in healthy controls.

### Molecular mimicry

The molecular mimicry hypothesis implies that an infectious agent elicits an immune response and that a cross-reaction occurs due to structural resemblance to human proteins [43-45]. To test the possibility of this hypothesis for the antigens assembled in our sets, we analyzed the prevalence of protein domains in general and the occurrence of ancient protein domains. In addition, we carried out a BLAST analysis of human proteins against complete sequenced organisms.

#### No general enriched protein domains, but single occurrences of Zinc finger and RNA recognition motifs

**Figure 3:**
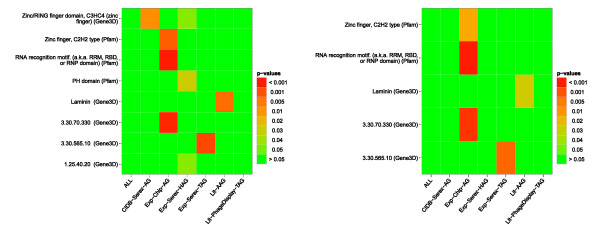
**Heat maps showing the significantly enriched protein domains**. Heat maps showing the significantly enriched protein domains compared to the ProteinCodingGenes reference set (left-hand side) or compared to the ProteinCodingGenesLongerExons reference set (right-hand side). ALL: union of all antigen sets; CIDB-Serex-AG: retrieved from the Cancer Immunome Database (SEREX method); Exp-Chip-AG: mixed antigens (tumor/non-tumor diseases) identified by protein macroarray; Exp-Serex-HAG: natural occurring autoantigens (SEREX method); Exp-Serex-TAG: tumor antigens (SEREX method); Lit-AAG: autoimmune antigens collected by literature search; Lit-PhageDisplay-TAG: tumor antigens identified by Phage Display experiments collected by literature search

To search for prevalence of protein domains, we subjected our data sets to an ORA for Gene3D (CATH) and Pfam domains in a first experiment. CATH is a database of manually derived structural domains from the Protein Data Bank (PDB) [46]. These domains are hierarchically organized according to topology, homology, and conservation [47]. Since CATH annotations are available for a small number of human proteins, we also used the CATH domain annotation generated by Gene3D [48]. Here, we extracted the CATH domains deposited in the Gene3D database v5.2.0. The Pfam database consists of conserved protein families and domains [49]. We used Pfam-A, which consists of high quality, manually generated families. As described previously, we considered only subcategories (in this case: domains) that appear in at least 5% of the annotated proteins of a data set to identify protein domains that occur frequently among antigens. Overall, only a few of several thousand analyzed domains meet the 5% threshold in our data sets. These results indicate that domains do not play a general role in the immunogenicity of antigens. However, we found evidence for an enrichment of Zinc finger motifs. In detail, antigens that we identified in tumor and non-tumor diseases (Exp-Chip-AG) showed enrichment for protein domains with a Zinc finger motif or an RNA recognition motif. Normal autoantigens (Exp-SEREX-HAG) and antigens derived from the Cancer Immunome Database (CIDB-Serex-AG) are enriched in a CATH domain named 'Zinc/RING finger domain'. The results are summarized in Figure 3. Since Zinc fingers as DNA-binding motifs are often found in transcription factors, these findings are consistent with the notion that proteins, which are part of cellular structures show an increased likelihood to become immunogenic.

#### Enrichment of ancient protein domains indicate that immunogenic proteins are evolutionary conserved

**Figure 4:**
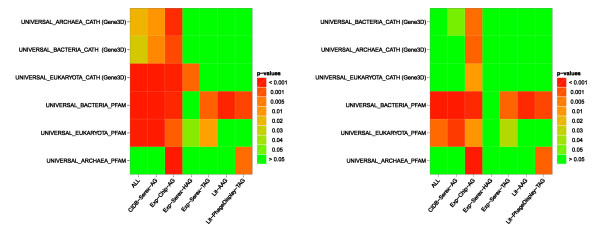
**Heat maps summarizing the enriched universal protein domains in the considered data sets**. Heat maps summarizing the enriched universal protein domains in the considered data sets compared to the ProteinCodingGenes reference set (left-hand side) or compared to the ProteinCodingGenesLongerExons reference set (right-hand side). ALL: union of all antigen sets; CIDB-Serex-AG: retrieved from the Cancer Immunome Database (SEREX method); Exp-Chip-AG: mixed antigens (tumor/non-tumor diseases) identified by protein macroarray; Exp-Serex-HAG: natural occurring autoantigens (SEREX method); Exp-Serex-TAG: tumor antigens (SEREX method); Lit-AAG: autoimmune antigens collected by literature search; Lit-PhageDisplay-TAG: tumor antigens identified by Phage Display-experiments collected by literature search

In a second experiment, we analyzed whether our antigen data sets contain predominantly 'ancient' protein families. To this end, we considered all species of the three kingdoms of life that are contained in the list of completely sequenced and published genomes from the Genomes OnLine Database (GOLD) v3.0 http://www.genomesonline.org/ and that are annotated with at least 150 protein domains. We obtained a similar distribution of organisms for CATH and Pfam domains and on average 450 different domains per organism. For each kingdom, we extracted all domains that occur in at least 70% of the species resulting in three sets of domains (BACTERIA, ARCHAEA, EUKARYOTA). For each kingdom and the two categories CATH and Pfam, we mapped the domains to their corresponding genes resulting in six UNIVERSAL sets containing genes with 'ancient' domains, e.g., UNIVERSAL_BACTERIA_CATH. To test the hypothesis whether our antigen sets are enriched with genes containing 'ancient' domains in comparison to our two reference sets, we performed ORAs for each of the collected UNIVERSAL sets. The following antigen sets show an enrichment of nearly all tested UNIVERSAL classes for the first reference set: The antigen set that contains all antigens (ALL), the set derived from the Cancer Immunome Database (CIDB-Serex-AG), and the set identified by our studies on tumor and non-tumor diseases (Exp-Chip-AG). Thus, the analysis of ancient protein domains indicates that immunogenic proteins are evolutionary conserved. The least number of enriched antigen sets was found for Archaea that show vast difference in their genetic makeup not only in comparison to Eukaryota, but also to Bacteria. Our results provide no evidence that ancient proteins are specifically enriched in normal autoantigens (Exp-SEREX-HAG). In summary, evolutionary conservation appears to be a feature of autoantigens in general. The results of this analysis are displayed in Figure 4.

#### Sequence similarities of autoantigens to proteins in other species support the mimicry hypothesis

**Figure 5:**
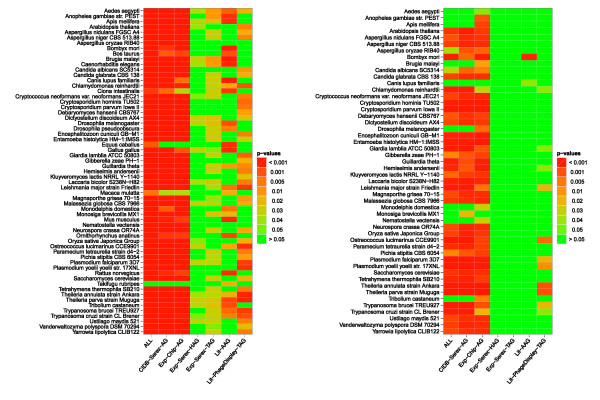
**Heat maps summarizing the enriched eukaryotic organisms in the considered data sets**. Heat maps summarizing the enriched eukaryotic organisms in the considered data sets compared to the ProteinCodingGenes reference set (left-hand side) or compared to the ProteinCodingGenesLongerExons reference set (right-hand side). ALL: union of all antigen sets; CIDB-Serex-AG: retrieved from the Cancer Immunome Database (SEREX method); Exp-Chip-AG: mixed antigens (tumor/non-tumor diseases) identified by protein macroarray; Exp-Serex-HAG: natural occurring autoantigens (SEREX method); Exp-Serex-TAG: tumor antigens (SEREX method); Lit-AAG: autoimmune antigens collected by literature search; Lit-PhageDisplay-TAG: tumor antigens identified by Phage Display experiments collected by literature search

As a third test, we explored whether our antigen sets have more similar sequences in other organisms than the reference set using the Basic Local Alignment Search Tool (BLAST) [50]. BLAST is a well-established method for finding local sequence similarities of a search pattern a database of sequences. The BLAST analysis has the advantage that we can find local sequence similarities that do not have to involve pre-defined functional domains, but that may also be candidates for eliciting immune responses via molecular mimicry. We performed a BLAST analysis of about 21000 human protein sequences against the protein sequences from RefSeq release 30 (including sequences from 5395 different organisms). In brief, we extracted for each of the human proteins the BLAST hits that had at least a similarity score of 100 and at most an E-value of 0.001. To retrieve the information to which kingdom of life these hits belong, we mapped the hits to their corresponding organisms. If a human protein had at least one BLAST hit in a specific organism, we added this human protein to the hit list of 'similar' proteins for this organism. For excluding hits in not completely sequenced organisms, we filtered the results using the list of completely sequenced and published genomes from GOLD. With the hit lists of 'similar' human proteins we performed an ORA for each organism comparing our antigen sets to our reference set. In summary, we found 61 of 82 tested eukaryotes, 437 of 447 organisms of the kingdom Bacteria, and 39 of 39 tested species from the group Archaea significantly enriched in at least one of our antigen sets. In the following, we will briefly discuss the results for the enriched eukaryotes (Figure 5) and the first reference set PCG. In analogy to the findings for the universal protein domains, we observe that the eukaryotic organisms are predominantly enriched for our data sets. The Exp-Serex-HAG set shows the lowest number of enriched organisms, followed by the Lit-AAG, Lit-PhageDisplay-TAG, and Exp-Serex-TAG set. The remaining sets present almost a uniform image of enriched organisms. Taking a closer look at the types of organisms included in Figure 5, we find well-known representatives of parasites. In addition, these parasites had most often the lowest p-values for our different data sets, e.g., Theileria parva strain Muguga, Theileria annulata strain Ankara, Plasmodium falciparum 3D7, Plasmodium yoelii yoelii str. 17XNL, Cryptosporidium parvum Iowa II, Entamoeba histolytica HM-1:IMSS, Cryptosporidium hominis, and Brugia malayi to mention the most important of these parasites. Taken together, these results show that a certain similarity to proteins of microbes exists and molecular mimicry may play a crucial role in the immunogenicity of antigens.

Considering the analysis with the second reference set PCGLE, the two large test sets CIDB-Serex-AG and Ex-Chip-AG that have also a significantly shorter mean exon length than the set PCGLE confirm basically the observations discussed above. However, the results obtained with the second reference set indicate that molecular mimicry might play a more important role for tumor antigens.

## Discussion

Multiple reasons may account for the immunogenicity of antigens including mutations, overexpression [51], alternative splicing [14,20], expression of fetal proteins in adult tissue, differential post-translational modifications, altered processes of apoptosis and necrosis, single nucleotide polymorphisms (SNPs), specific sequence motifs, epitope spreading, molecular mimicry, and differential cellular localization [12,13]. There is none or only circumstantial experimental evidence for almost all of these hypotheses. Likewise, there was virtually no information whether any of the above listed features can be preferentially found with a particular group of antigens like natural-occurring, autoimmunity-associated and tumor-associated antigens.

For most considered antigen sets, we found enrichment of sequence-based properties including coiled-coil motifs, ELR motifs, and Zinc finger DNA-binding motifs. Some of these features have been lately proposed as important for the immunogenicity of autoantigens such as coiled-coils domains [52] or Granzyme B cleavage sites [53]. ELR motifs are supposed to be functional domains with chemotactic properties that play a role in CXC chemokines [39]. Since CXC chemokines containing ELR motifs are important for the activation of leukocytes that take part in phagocytosis of microbes and foreign antigens they have the ability to activate the immune system [54]. An overview about motifs important for the autoantibody repertoire is given by Plotz [44]. Although both these previously reported data and our data provide evidence for a decisive role of sequence motifs in the immunogenicity of autoantigens, it is important to bear in mind that some features like cleavage sites and the coiled-coils are solely based on predictions and still await experimental confirmation [37,38].

Beside sequence-based properties, we found that antigens in our data sets are frequently enriched for the characteristics of protein binding and DNA or RNA binding. Many of the immunogenic proteins are also part of or are associated with ribosomes and spliceosomes. These results indicate that complex cellular structures and especially ribosomes are frequently targets of autoantibodies. This is in keeping with the particle hypothesis of Tan and Hardin that suggested that autoantibodies frequently do not target single proteins but cell organelles [55,56]. This hypothesis does, however, not answer the question why antibodies are frequently directed against complex cellular structures like ribosomes. The answer may be provided by another result of our study showing increased similarity of the proteins in the antigen sets to proteins of other species. Due to this increased similarity proteins may have a higher probability to become immunogenic than proteins that are more specific for human. The reasoning behind that idea is as follows. The adaptive immune system must be flexible enough to detect a wide range of possible pathogenic targets, even those that are similar to self-antigens. However, this flexibility comes with the risk of autoimmune diseases [57]. In detail, an infectious agent elicits an immune response and a cross-reaction occurs due to structural resemblance to a human protein. An example of an infection with a subsequent cross-reactivity against a self-antigen is the gastric autoimmunity that is associated with *Helicobacter pylori *antigens [58]. Since many of the complex cellular structures like ribosomes are evolutionary highly conserved, the molecular mimicry hypothesis may explain why these structures are preferentially targeted by autoantigens. In particular, for ribosomes that often had the most significant p-values, it is conceivable that their immunogenicity may be advanced by attached peptides in statu nascendi (not completely folded or in conformational transition).

In addition to general features identified for antigens of our sets, there are some characteristics that appear to be more specific for single antigen sets. We found evidence for an enrichment of both (non)-synonymous SNPs and synonymous SNPs in antigens of autoimmune diseases. Antigens of autoimmune diseases are also enriched for GO terms 'extracellular region/space', 'plasma membrane part', 'vesicle', and 'secretory granule' showing a propensity for secretion and extracellular location. This may contribute to the stimulation of an immune reaction. Notably extracellular targets of autoantibodies in autoimmune diseases are often directly linked to the pathogenesis of the disease [59]. In the majority of cases, it may not be feasible to discern between a general immunogenic feature and a feature specific for natural-occurring, autoimmunity-associated and tumor-associated antigens. Some of the above features that are potentially found in all antigens may preferentially be targeted by autoantibody in the course of tumor development. Proteins of cancer cells can stimulate an immune reaction by necrotic processes or defective apoptosis. While apoptosis is normally an anti-inflammatory process with cell debris removed by phagocytic cells, an abnormal apoptosis could lead to APC (Antigen-Presenting Cell) activation and presentation of self-antigens. Necrosis is in general a pro-inflammatory process that occurs during tumor growth exposing the contents of the cell to the immune system. Proteins with features identified in this study, e.g. high similarity to foreign proteins, are likely to be more susceptible to elicit immune responses in the course of tumor related processes than proteins without these features.

Interestingly, several of the features identified as pro-immunogenic in this study, are found with proteins that are known to play a role in tumor development. A significant portion of the immunogenic antigen sets showed enrichment for Zinc-finger motifs that are often found in transcription factors. These are frequently over-expressed in cancer driving the proliferation in tumor cells. We also found many immunogenic proteins involved in ribosomes that are discussed to play an active role in tumorigenesis [60,61]. This finding is in line with a previous study also reporting that tumor antigens often play a crucial role in carcinogenesis [13]. Our findings concerning the increased exon lengths of our antigen sets also support the "stimulation-responsive splicing" model of Yang et al. [20]. Genes with more exons can probably create more splice variants by different combinations of their exons, which may lead to the presentation of untolerized epitopes to the immune system. In addition, specific sequence features may be responsible for making those autoantigens more prone for being processed by the immune system (GrB cleavage sites, coiled-coils motifs) or increase the probability for being recognized as foreign (SNPs, mutations).

A crucial point that influences the results of all performed analyses is the selection of antigen sets that were used in this work. The properties of the antigen sets seem to be at least in part dependent on their experimental technique as indicated by the fact that the antigens derived from the SEREX method and the protein chip often built separate clusters in our analyses. Furthermore, most of the considered antigens were detected by few sera only and the mode of detecting positive antigen-antibody reactions during isolation is commonly error-prone. Taking these factors into consideration, we were still able to gain new insights in a highly complex field of research.

## Conclusions

Autoantibodies against self-antigens have been associated not only with autoimmune diseases, but also with cancer and are even found in healthy individuals. Whilst disease associated antigens are already applied as biomarkers and tumor antigens are studied as putative vaccines in cancer immunotherapy, the mechanism causing the autoantibody response and the loss of self-tolerance remains elusive for the majority of the known immunogenic antigens. To deepen the understanding of autoantibody responses, we asked whether natural-occurring, autoimmunity-associated and tumor-associated antigens have structural or biological features in common. To this end, we have carried out the most comprehensive in-silicio study of different groups of autoantigens including large antigen sets identified by our groups combined with publicly available sets. Our results indicate that autoimmunogenic proteins are evolutionary conserved and that molecular mimicry might also play a central role. Taken together, we provided further indications for differences and similarities in tumor antigens and autoantigens. However, the picture that emerged is by far not complete. More effort and research will be necessary to deepen our understanding of the immunogenicity of autoantigens.

## Methods

### Statistical Methods

In order to assess the enrichment of gene sets, we used the statistical methods integrated in GeneTrail [33]. In this work, we applied two statistical tests, the so-called Over-Representation Analysis (ORA) using the hypergeometric distribution test and the Wilcoxon-Mann-Whitney (WMW) test [62]. We applied the ORA in this work for binary biological categories (e.g. a gene can either belong to a certain pathway or not). For non-binary categories (e.g. number of SNPs), we employed the unpaired one-tailed WMW test to explore if the probability distribution for the values of the test set genes is shifted to the right of the distribution for the values of the reference set genes. The computed significance values for both statistical tests are adjusted by applying the Benjamini-Hochberg approach [63].

#### Data Sets

The antigen sets used in this work stem either from our experiments (SEREX, protein arrays), from databases or from literature search. The different data sets were named as follows: First, we indicated the source ('Lit' for collected from literature, 'CIDB' for the Cancer Immunome Database http://ludwig-sun5.unil.ch/CancerImmunomeDB/, 'Exp' for our own experimental data), second the experimental method (e.g. 'Serex', 'Chip', and 'PhageDisplay'), and third the type of antigens contained in the data set (AG for all antigens, AAG for autoimmune antigens, HAG for antigens occurring in healthy-persons, TAG for tumor antigens).

The antigen set CIDB-Serex-AG was extracted from CIDB in February 2009 and contains 1471 antigen encoding genes. The antigens were originally detected by SEREX approach in various cancers [64]. The sequences and identities were deposited in the CIDB database.

The antigen sets Exp-Serex-HAG and Exp-Serex-TAG were derived from our experiments also using SEREX. Exp-Serex-HAG contains 86 known genes that were detected with sera of healthy persons, and Exp-Serex-TAG contains 74 antigens that were reactive with sera of different cancer patients including patients with meningioma, glioma or lung cancer [7,8,65].

We also identified the set Exp-Chip-AG containing 298 antigens using a protein macroarray that contained proteins derived from a recombinant human fetal brain library [66]. The library that contained 38,000 E. coli clones was screened with 30 serum pools of patients with different tumor and non tumor diseases including prostate cancer, lung cancer, meningioma, glioma, wilms tumor, neuroblastoma, morbus crohn, colitis ulcerosa, stroke and benign prostate hyperplasia and healthy controls [9,10,67,68]. All antigens that were positive for at least one serum pool were included in our analysis.

Two data sets were derived from literature search. The Lit-PhageDisplay-TAG set contains 84 tumor antigens that were isolated with the Phage Display library method. The antigen set Lit-AAG that is online available contains 348 genes associated with autoimmune diseases http://www.wiley-vch.de/contents/jc_2040/2005/25481_s.pdf. This set was initially collected to analyze the occurrences of SNPs (single nucleotide polymorphisms) in autoantigens [36].

The ALL set contains the union of all antigens of the data sets Lit-AAG, Exp-Serex-HAG, Exp-Chip-AG, Exp-Serex-TAG, CIDB-Serex-AG, and Lit-PhageDisplay-TAG. This set is to find patterns prevalent to all antigens.

As first reference set - termed ProteinCodingGenes -, we used all human protein coding genes excluding the above-mentioned antigens (human protein coding genes minus genes in the ALL set). In order to exclude a bias that could be introduced by the exon lengths of the genes in the sets, we performed the analyses with a second reference set - termed ProteinCodingGenesLongerExons - that consists of the genes of the first reference set whose exon lengths were larger than 3100 nucleotides. This results in a reference set that contains genes having significantly larger exon lengths or do not differ significantly when compared to the different antigen sets. The different data sets are summarized in Table 1. Some representative examples of autoantigens found in our data sets are listed in Table 2.

**Table 2:** Representative antigens in our data sets.

autoimmunity-associated antigens		natural-occurring antigens	tumor-associated antigens
systemic	organ-specific	SALL2 LSM2 METTL13	BRCA2 CALM1 TP53
SSB TRIM21 TROVE2 CAST SNRNP70 VIM SSSCA1 XRCC6	GAD1 PTPRN TG	PMS1 SMG1 RAB11FIP4	CTAG2 CTAG1A BRAP
SSSCA1 XRCC6 SSNA1 XRCC5	SLC25A16	CRK USP31	SART1 SSX2 IGF2BP2

If not mentioned otherwise we performed the analyses for all antigen sets using GeneTrail [33] with the following parameters: significance level: 0.05; minimum number of genes in a subcategory: 2; p-value computation: FDR correction; reference set: ProteinCodingGenes. When performing an Over-Representation Analysis (ORA), we filtered the results afterwards for significantly enriched subcategories where at least 5% of the genes of the test set had an annotation for the considered category. Hereby, we focused on subcategories that show prevalence in our antigen sets.

## Competing interests

The authors declare that they have no competing interests.

## Authors' contributions

CB implemented and performed the computational analyses, analyzed and interpreted the data, and wrote the manuscript. NL, PL, CH, JH performed the experiments for detecting antigens and collected antigen data from literature. NL also participated in writing the manuscript. AK participated in the analysis of the data. EM and HPL conceived of the study, participated in interpreting the data and writing of the manuscript. All authors read and approved the final manuscript.

## Supplementary Material

Additional file 1 **Figure S1: Overview of all significant GO categories**. Heat maps showing the significantly enriched GO terms in the considered data sets compared to the ProteinCodingGenes reference set (left-hand side) or compared to the ProteinCodingGenesLongerExons reference set (right-hand side). ALL: union of all antigen sets; CIDB-Serex-AG: retrieved from the Cancer Immunome Database (SEREX method); Exp-Chip-AG: mixed antigens (tumor/non-tumor diseases) identified by protein macroarray; Exp-Serex-HAG: natural occurring autoantigens (SEREX method); Exp-Serex-TAG: tumor antigens (SEREX method); Lit-AAG: autoimmune antigens collected by literature search; Lit-PhageDisplay-TAG: tumor antigens identified by Phage Display experiments collected by literature search. Click here for file

Additional file 2 **Figure S2: Overview of all significant KEGG categories**. Heat maps showing the significantly enriched KEGG pathways in the considered data sets compared to the ProteinCodingGenes reference set (left-hand side) or compared to the ProteinCodingGenesLongerExons reference set (right-hand side). ALL: union of all antigen sets; CIDB-Serex-AG: retrieved from the Cancer Immunome Database (SEREX method); Exp-Chip-AG: mixed antigens (tumor/non-tumor diseases) identified by protein macroarray; Exp-Serex-HAG: natural occurring autoantigens (SEREX method); Exp-Serex-TAG: tumor antigens (SEREX method); Lit-AAG: autoimmune antigens collected by literature search; Lit-PhageDisplay-TAG: tumor antigens identified by Phage Display experiments collected by literature search. Click here for file
