# Supplementary material for: Immunogenicity of autoantigens
Source: BMC Genomics. 2011 Jul 4;12:340. doi: 10.1186/1471-2164-12-340 (PMC3149588; doi:10.1186/1471-2164-12-340)
Supplement: Additional file 1 — Figure S1: Overview of all significant GO categories. Heat maps showing the significantly enriched GO terms in the considered data sets compared to the ProteinCodingGenes reference set (left-hand side) or compared to the ProteinCodingGenesLongerExons reference set (right-hand side). ALL: union of all antigen sets; CIDB-Serex-AG: retrieved from the Cancer Immunome Database (SEREX method); Exp-Chip-AG: mixed antigens (tumor/non-tumor diseases) identified by protein macroarray; Exp-Serex-HAG: natural occurring autoantigens (SEREX method); Exp-Serex-TAG: tumor antigens (SEREX method); Lit-AAG: autoimmune antigens collected by literature search; Lit-PhageDisplay-TAG: tumor antigens identified by Phage Display experiments collected by literature search. [file 1471-2164-12-340-S1.PDF]

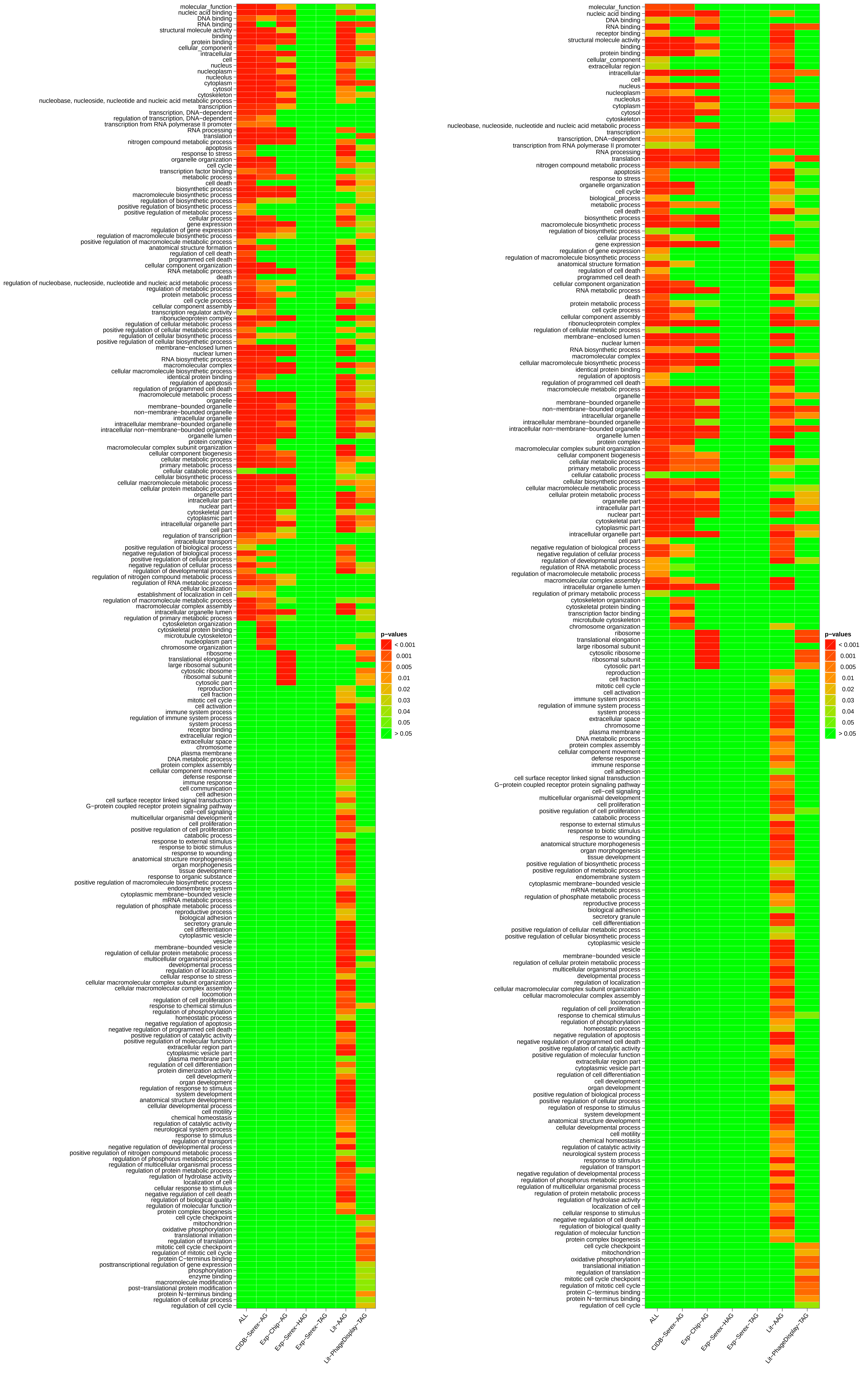

molecular_function
nucleic acid binding
DNA binding
RNA binding
structural molecule activity
binding
protein binding
cellular_component
intracellular
cell
nucleus
nucleoplasm
nucleolus
cytoplasm
cytosol
cytoskeleton
nucleobase, nucleoside, nucleotide and nucleic acid metabolic process
transcription
transcription, DNA−dependent
regulation of transcription, DNA−dependent
transcription from RNA polymerase II promoter
RNA processing
translation
nitrogen compound metabolic process
apoptosis
response to stress
organelle organization
cell cycle
transcription factor binding
metabolic process
cell death
biosynthetic process
macromolecule biosynthetic process
regulation of biosynthetic process
positive regulation of biosynthetic process
positive regulation of metabolic process
cellular process
gene expression
regulation of gene expression
regulation of macromolecule biosynthetic process
positive regulation of macromolecule metabolic process
anatomical structure formation
regulation of cell death
programmed cell death
cellular component organization
RNA metabolic process
death
regulation of nucleobase, nucleoside, nucleotide and nucleic acid metabolic process
regulation of metabolic process
protein metabolic process
cell cycle process
cellular component assembly
transcription regulator activity
ribonucleoprotein complex
regulation of cellular metabolic process
positive regulation of cellular metabolic process
regulation of cellular biosynthetic process
positive regulation of cellular biosynthetic process
membrane−enclosed lumen
nuclear lumen
RNA biosynthetic process
macromolecular complex
cellular macromolecule biosynthetic process
identical protein binding
regulation of apoptosis
regulation of programmed cell death
macromolecule metabolic process
organelle
membrane−bounded organelle
non−membrane−bounded organelle
intracellular organelle
intracellular membrane−bounded organelle
intracellular non−membrane−bounded organelle
organelle lumen
protein complex
macromolecular complex subunit organization
cellular component biogenesis
cellular metabolic process
primary metabolic process
cellular catabolic process
cellular biosynthetic process
cellular macromolecule metabolic process
cellular protein metabolic process
organelle part
intracellular part
nuclear part
cytoskeletal part
cytoplasmic part
intracellular organelle part
cell part
regulation of transcription
intracellular transport
positive regulation of biological process
negative regulation of biological process
positive regulation of cellular process
negative regulation of cellular process
regulation of developmental process
regulation of nitrogen compound metabolic process
regulation of RNA metabolic process
cellular localization
establishment of localization in cell
regulation of macromolecule metabolic process
macromolecular complex assembly
intracellular organelle lumen
regulation of primary metabolic process
cytoskeleton organization
cytoskeletal protein binding
microtubule cytoskeleton
nucleoplasm part
chromosome organization
ribosome
translational elongation
large ribosomal subunit
cytosolic ribosome
ribosomal subunit
cytosolic part
reproduction
cell fraction
mitotic cell cycle
cell activation
immune system process
regulation of immune system process
system process
receptor binding
extracellular region
extracellular space
chromosome
plasma membrane
DNA metabolic process
protein complex assembly
cellular component movement
defense response
immune response
cell communication
cell adhesion
cell surface receptor linked signal transduction
G−protein coupled receptor protein signaling pathway
cell−cell signaling
multicellular organismal development
cell proliferation
positive regulation of cell proliferation
catabolic process
response to external stimulus
response to biotic stimulus
response to wounding
anatomical structure morphogenesis
organ morphogenesis
tissue development
response to organic substance
positive regulation of macromolecule biosynthetic process
endomembrane system
cytoplasmic membrane−bounded vesicle
mRNA metabolic process
regulation of phosphate metabolic process
reproductive process
biological adhesion
secretory granule
cell differentiation
cytoplasmic vesicle
vesicle
membrane−bounded vesicle
regulation of cellular protein metabolic process
multicellular organismal process
developmental process
regulation of localization
cellular response to stress
cellular macromolecular complex subunit organization
cellular macromolecular complex assembly
locomotion
regulation of cell proliferation
response to chemical stimulus
regulation of phosphorylation
homeostatic process
negative regulation of apoptosis
negative regulation of programmed cell death
positive regulation of catalytic activity
positive regulation of molecular function
extracellular region part
cytoplasmic vesicle part
plasma membrane part
regulation of cell differentiation
protein dimerization activity
cell development
organ development
regulation of response to stimulus
system development
anatomical structure development
cellular developmental process
cell motility
chemical homeostasis
regulation of catalytic activity
neurological system process
response to stimulus
regulation of transport
negative regulation of developmental process
positive regulation of nitrogen compound metabolic process
regulation of phosphorus metabolic process
regulation of multicellular organismal process
regulation of protein metabolic process
regulation of hydrolase activity
localization of cell
cellular response to stimulus
negative regulation of cell death
regulation of biological quality
regulation of molecular function
protein complex biogenesis
cell cycle checkpoint
mitochondrion
oxidative phosphorylation
translational initiation
regulation of translation
mitotic cell cycle checkpoint
regulation of mitotic cell cycle
protein C−terminus binding
posttranscriptional regulation of gene expression
phosphorylation
enzyme binding
macromolecule modification
post−translational protein modification
protein N−terminus binding
regulation of cellular process
regulation of cell cycle
ALL
CIDB−Serex−AG
Exp−Chip−AG
Exp−Serex−HAG
Exp−Serex−TAG
Lit−AAG
Lit−PhageDisplay−TAG
p−values
< 0.001
0.001
0.005
0.01
0.02
0.03
0.04
0.05
> 0.05
molecular_function
nucleic acid binding
DNA binding
RNA binding
receptor binding
structural molecule activity
binding
protein binding
cellular_component
extracellular region
intracellular
cell
nucleus
nucleoplasm
nucleolus
cytoplasm
cytosol
cytoskeleton
nucleobase, nucleoside, nucleotide and nucleic acid metabolic process
transcription
transcription, DNA−dependent
transcription from RNA polymerase II promoter
RNA processing
translation
nitrogen compound metabolic process
apoptosis
response to stress
organelle organization
cell cycle
biological_process
metabolic process
cell death
biosynthetic process
macromolecule biosynthetic process
regulation of biosynthetic process
cellular process
gene expression
regulation of gene expression
regulation of macromolecule biosynthetic process
anatomical structure formation
regulation of cell death
programmed cell death
cellular component organization
RNA metabolic process
death
protein metabolic process
cell cycle process
cellular component assembly
ribonucleoprotein complex
regulation of cellular metabolic process
membrane−enclosed lumen
nuclear lumen
RNA biosynthetic process
macromolecular complex
cellular macromolecule biosynthetic process
identical protein binding
regulation of apoptosis
regulation of programmed cell death
macromolecule metabolic process
organelle
membrane−bounded organelle
non−membrane−bounded organelle
intracellular organelle
intracellular membrane−bounded organelle
intracellular non−membrane−bounded organelle
organelle lumen
protein complex
macromolecular complex subunit organization
cellular component biogenesis
cellular metabolic process
primary metabolic process
cellular catabolic process
cellular biosynthetic process
cellular macromolecule metabolic process
cellular protein metabolic process
organelle part
intracellular part
nuclear part
cytoskeletal part
cytoplasmic part
intracellular organelle part
cell part
negative regulation of biological process
negative regulation of cellular process
regulation of developmental process
regulation of RNA metabolic process
regulation of macromolecule metabolic process
macromolecular complex assembly
intracellular organelle lumen
regulation of primary metabolic process
cytoskeleton organization
cytoskeletal protein binding
transcription factor binding
microtubule cytoskeleton
chromosome organization
ribosome
translational elongation
large ribosomal subunit
cytosolic ribosome
ribosomal subunit
cytosolic part
reproduction
cell fraction
mitotic cell cycle
cell activation
immune system process
regulation of immune system process
system process
extracellular space
chromosome
plasma membrane
DNA metabolic process
protein complex assembly
cellular component movement
defense response
immune response
cell adhesion
cell surface receptor linked signal transduction
G−protein coupled receptor protein signaling pathway
cell−cell signaling
multicellular organismal development
cell proliferation
positive regulation of cell proliferation
catabolic process
response to external stimulus
response to biotic stimulus
response to wounding
anatomical structure morphogenesis
organ morphogenesis
tissue development
positive regulation of biosynthetic process
positive regulation of metabolic process
endomembrane system
cytoplasmic membrane−bounded vesicle
mRNA metabolic process
regulation of phosphate metabolic process
reproductive process
biological adhesion
secretory granule
cell differentiation
positive regulation of cellular metabolic process
positive regulation of cellular biosynthetic process
cytoplasmic vesicle
vesicle
membrane−bounded vesicle
regulation of cellular protein metabolic process
multicellular organismal process
developmental process
regulation of localization
cellular macromolecular complex subunit organization
cellular macromolecular complex assembly
locomotion
regulation of cell proliferation
response to chemical stimulus
regulation of phosphorylation
homeostatic process
negative regulation of apoptosis
negative regulation of programmed cell death
positive regulation of catalytic activity
positive regulation of molecular function
extracellular region part
cytoplasmic vesicle part
regulation of cell differentiation
cell development
organ development
positive regulation of biological process
positive regulation of cellular process
regulation of response to stimulus
system development
anatomical structure development
cellular developmental process
cell motility
chemical homeostasis
regulation of catalytic activity
neurological system process
response to stimulus
regulation of transport
negative regulation of developmental process
regulation of phosphorus metabolic process
regulation of multicellular organismal process
regulation of protein metabolic process
regulation of hydrolase activity
localization of cell
cellular response to stimulus
negative regulation of cell death
regulation of biological quality
regulation of molecular function
protein complex biogenesis
cell cycle checkpoint
mitochondrion
oxidative phosphorylation
translational initiation
regulation of translation
mitotic cell cycle checkpoint
regulation of mitotic cell cycle
protein C−terminus binding
protein N−terminus binding
regulation of cell cycle
ALL
CIDB−Serex−AG
Exp−Chip−AG
Exp−Serex−HAG
Exp−Serex−TAG
Lit−AAG
Lit−PhageDisplay−TAG
p−values
< 0.001
0.001
0.005
0.01
0.02
0.03
0.04
0.05
> 0.05
