# Supplementary material for: Immunogenicity of autoantigens
Source: BMC Genomics. 2011 Jul 4;12:340. doi: 10.1186/1471-2164-12-340 (PMC3149588; doi:10.1186/1471-2164-12-340)
Supplement: Additional file 2 — Figure S2: Overview of all significant KEGG categories. Heat maps showing the significantly enriched KEGG pathways in the considered data sets compared to the ProteinCodingGenes reference set (left-hand side) or compared to the ProteinCodingGenesLongerExons reference set (right-hand side). ALL: union of all antigen sets; CIDB-Serex-AG: retrieved from the Cancer Immunome Database (SEREX method); Exp-Chip-AG: mixed antigens (tumor/non-tumor diseases) identified by protein macroarray; Exp-Serex-HAG: natural occurring autoantigens (SEREX method); Exp-Serex-TAG: tumor antigens (SEREX method); Lit-AAG: autoimmune antigens collected by literature search; Lit-PhageDisplay-TAG: tumor antigens identified by Phage Display experiments collected by literature search. [file 1471-2164-12-340-S2.PDF]

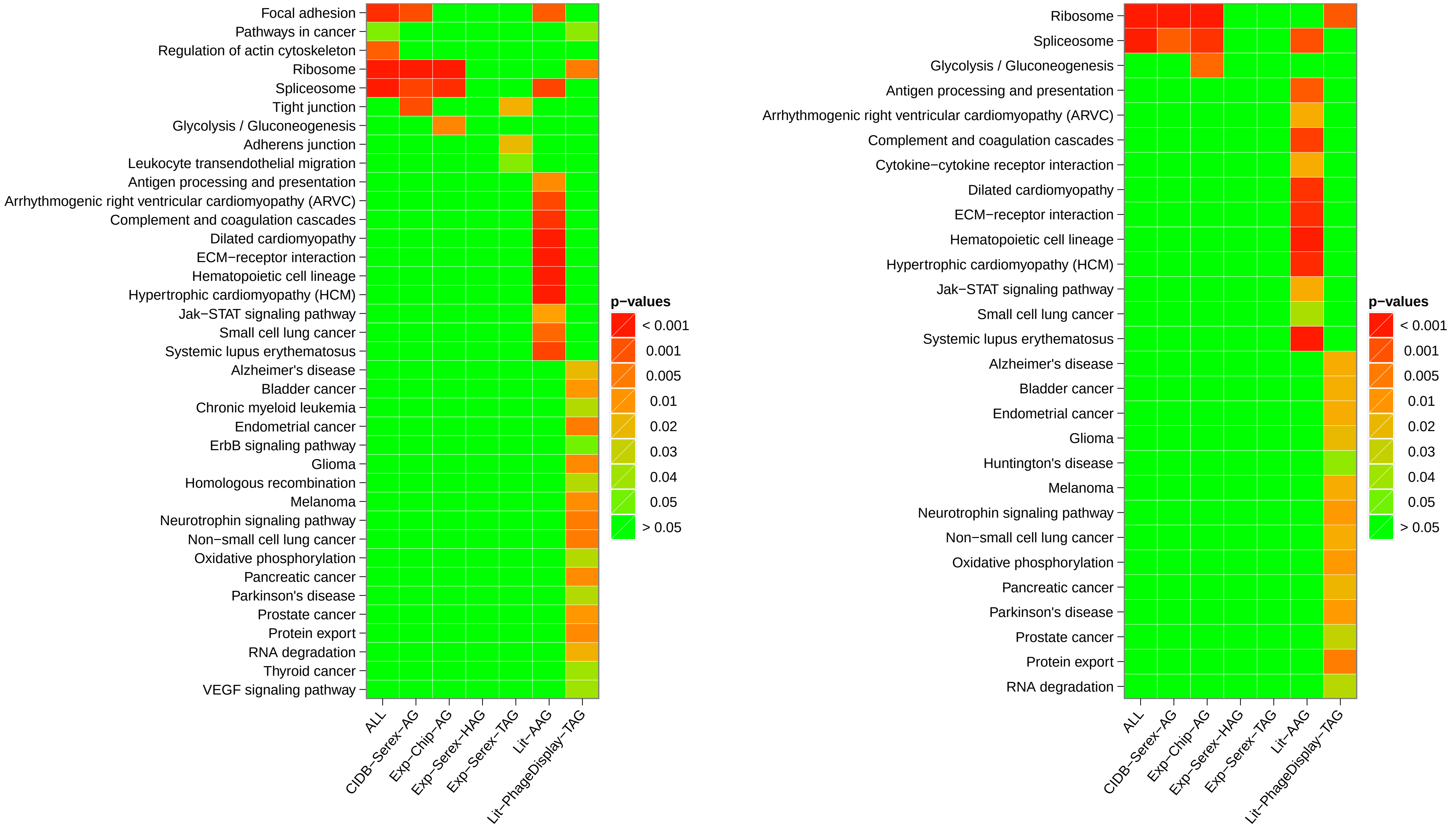
Focal adhesion
Pathways in cancer
Regulation of actin cytoskeleton
Ribosome
Spliceosome
Tight junction
Glycolysis / Gluconeogenesis
Adherens junction
Leukocyte transendothelial migration
Antigen processing and presentation
Arrhythmogenic right ventricular cardiomyopathy (ARVC)
Complement and coagulation cascades
Dilated cardiomyopathy
ECM−receptor interaction
Hematopoietic cell lineage
Hypertrophic cardiomyopathy (HCM)
Jak−STAT signaling pathway
Small cell lung cancer
Systemic lupus erythematosus
Alzheimer's disease
Bladder cancer
Chronic myeloid leukemia
Endometrial cancer
ErbB signaling pathway
Glioma
Homologous recombination
Melanoma
Neurotrophin signaling pathway
Non−small cell lung cancer
Oxidative phosphorylation
Pancreatic cancer
Parkinson's disease
Prostate cancer
Protein export
RNA degradation
Thyroid cancer
VEGF signaling pathway
ALL
CIDB−Serex−AG
Exp−Chip−AG
Exp−Serex−HAG
Exp−Serex−TAG
Lit−AAG
Lit−PhageDisplay−TAG
p−values
< 0.001
0.001
0.005
0.01
0.02
0.03
0.04
0.05
> 0.05
Ribosome
Spliceosome
Glycolysis / Gluconeogenesis
Antigen processing and presentation
Arrhythmogenic right ventricular cardiomyopathy (ARVC)
Complement and coagulation cascades
Cytokine−cytokine receptor interaction
Dilated cardiomyopathy
ECM−receptor interaction
Hematopoietic cell lineage
Hypertrophic cardiomyopathy (HCM)
Jak−STAT signaling pathway
Small cell lung cancer
Systemic lupus erythematosus
Alzheimer's disease
Bladder cancer
Endometrial cancer
Glioma
Huntington's disease
Melanoma
Neurotrophin signaling pathway
Non−small cell lung cancer
Oxidative phosphorylation
Pancreatic cancer
Parkinson's disease
Prostate cancer
Protein export
RNA degradation
ALL
CIDB−Serex−AG
Exp−Chip−AG
Exp−Serex−HAG
Exp−Serex−TAG
Lit−AAG
Lit−PhageDisplay−TAG
p−values
< 0.001
0.001
0.005
0.01
0.02
0.03
0.04
0.05
> 0.05
